# About Gondolas

**Published:** 1881-12

**Authors:** 


### ABOUT GONDOLAS.

It is related that recently one of the New York aldermen had an idea. Moved by its rarity, he hastened to lay it before his brother Solons. "Gentlemen," said he, "I think it would add to the attractiveness of Central Park if we were to import some gondolas—say a dozen—and place them in the lake." The idea was favorably received by all but one. He was the economist of the board, and in his veins ran some of the blood of Irish kings. He rose. "Gintlemen," he remarked, "the idea is a good wan, but I wud make an amindment. Why should we buy twelve av thim? It wud be a useless expinse. I make a motion that we buy two av thim—a male wan and a female wan. Thin, gintlemin, let nature take her coorse."—*Argonaut.*

